# Infection Dynamics of *Mycoplasma bovis* and Other Respiratory Mycoplasmas in Newly Imported Bulls on Italian Fattening Farms

**DOI:** 10.3390/pathogens9070537

**Published:** 2020-07-04

**Authors:** Salvatore Catania, Michele Gastaldelli, Eliana Schiavon, Andrea Matucci, Annalucia Tondo, Marianna Merenda, Robin A. J. Nicholas

**Affiliations:** 1 Istituto Zooprofilattico Sperimentale delle Venezie, viale Dell’Università 10, Legnaro (PD371735, Italy; scatania@izsvenezie.it (S.C.); mgastaldelli@izsvenezie.it (M.G.); eschiavon@izsvenezie.it (E.S.); amatucci@izsvenezie.it (A.M.); atondo@izsvenezie.it (A.T.); mmerenda@izsvenezie.it (M.M.); 2 The Oaks, Nutshell Lane, Farnham, Surrey GU9 0HG, UK

**Keywords:** *Mycoplasma bovis*, bovine respiratory disease, cattle, prevalence

## Abstract

Italian beef production is mainly based on a feedlot system where calves are housed with mixed aged cattle often in conditions favourable to bovine respiratory disease (BRD). In Veneto, an indoor system is also used for imported bulls around 300–350 kg. Mycoplasmas, in particular *Mycoplasma bovis* and *Mycoplasma dispar*, contribute to BRD in young calves, but their role in the disease in older cattle has not been investigated. In this study, ten heads of cattle were selected from each of the 24 groups kept in 13 different farms. Bulls were sampled by nasal swabbing at 0, 15, and 60 days after arrival for *Mycoplasma* isolation. Identification was carried out by 16S-rDNA PCR followed by denaturing gradient gel electrophoresis. *M. bovis*, *M. dispar*, and *M. bovirhinis* were identified, and prevalence was analysed by mixed-effects logistic regression models. This showed that most bulls arrived free of *M. bovis*, but within two weeks, approximately 40% became infected, decreasing to 13% by the last sampling. In contrast, the prevalence of *M. dispar* was not dependent on time or seasonality, while *M. bovirhinis* only showed a seasonality-dependent trend. The Italian fattening system creates an ideal environment for infection with *M. bovis*, probably originating from previously stabled animals.

## 1. Introduction

Most European countries operate a feedlot system for male beef production where young calves, usually about one month old, are brought in mostly from dairy farms, then fattened to approximately 240 kg. In Italy, a mixed-age indoor system is also used, which involves the importation of bulls at 300–350 kg from other European countries. This system is mainly located in the Po Valley with the largest herds in the Veneto region [1] and accounts for approximately an 85% share of the beef market. On arrival, the bulls are placed indoors directly with cattle of different ages often sharing the same air space. The cattle are kept for approximately 6 months until they reach a target weight of approximately 650 kg. While relatively productive, the system is prone to severe outbreaks of bovine respiratory disease (BRD) caused by different pathogens, such as bovine viral diarrhoea (BVD) virus, para-influenza virus 3 (PI3), infectious bovine rhinotracheitis (IBR) virus, *Pasteurella*, *Mannheimia*, and mycoplasmas [2]. BRD is often exacerbated by overcrowding and poor ventilation and compounded by the heterogeneity of breeds and diverse origins of the cattle.

At least 30 different mycoplasmas have been isolated from cattle, of which only a few are considered pathogenic, notably *Mycoplasma bovis* and *M. dispar*, which can cause serious respiratory disease in young and adult animals, respectively [3]. Other mycoplasmas have a pathogenic impact on the reproductive system, such as *M. bovigenitalium* [4], while others, such as *M. bovis*, *M. californicum*, and *M. canadense*, are causes of or associated with mastitis [5]. *M. bovirhinis* is frequently isolated from the respiratory tract but is mostly considered to be non-pathogenic [6].

*M. bovis* has been identified as the major pathogen affecting young animals in northern Italy [2] and is suspected of being involved in disease in older livestock. For this reason, we decided to investigate the prevalence and epidemiology of *Mycoplasma* species in this specialised older cattle sector.

In this study, we used *Mycoplasma* isolation and species identification by 16S-rDNA PCR, followed by denaturing gradient gel electrophoresis (DGGE) to assess the prevalence of *M. bovis*, *M. dispar*, and *M. bovirhinis* in different batches of imported bulls stabled in Italian farms. Animals were sampled by nasal swabs at different times after arrival following a longitudinal experimental design. In addition to isolation, *M. bovis* presence was also determined by a specific PCR protocol.

## 2. Results

Of the 711 analysed nasal swabs, 485 (68.2%) were positive for species belonging to the *Mollicutes* class. The majority of the isolated organisms belonged to the species *M. bovirhinis* (283, 39.8%), *M. bovis* (136, 19.1%), *M. dispar* (86, 12.1%), and to species of the genus *Ureaplasma* (66, 9.3%) (Table S1). Approximately half of all isolated organisms were found in mixed cultures with other species of the *Mollicutes* class. In addition, *M. bovis* was detected in 276 swabs (approximately 39% of the total samples) by direct PCR in contrast to the 136 isolates (19% of the total samples) obtained by culture.

### 2.1. Analysis of Prevalence of Mollicutes Class Organisms

**Figure 1:**
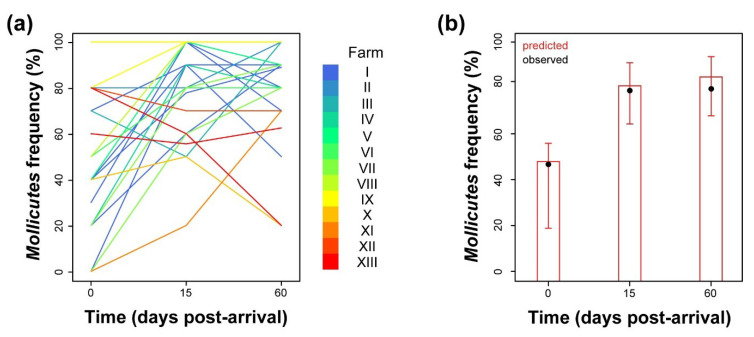
(**a**) Batch-related frequency of isolation of organisms belonging to the *Mollicutes* class analysed over time after arrival. Each line colour is depicted according to the identity of the stabling farm. (**b**) Model-predicted *Mollicutes* prevalence inferred at the population level over time post arrival (red). Observed mean prevalence values are depicted as solid black circles. Vertical lines correspond to the 95% CI of the predicted mean.

Isolates identified as belonging to the *Mollicutes* class largely varied in prevalence over time post-arrival and among the different bull batches and fattening farms (Figure 1a). 

However, at the population level, we could identify a clear, significant time-dependent trend (Tables S2 and S3) characterised by an initial prevalence value of incoming animals of approximately 48%, with a 95% confidence interval (95% CI) ranging from 30% to 67%. At 15 days post-arrival (p.a.), the estimated frequency of *Mollicutes*-positive animals significantly increased with an odds ratio of 4.6 (95% CI, 2.2–10.5; adjusted *p* = 0.003) to reach a plateau at approximately 81% (Figure 1b). No effects of the environmental conditions (variable “season”, see the paragraph in Materials and Methods) on predicted prevalence were observed (Table S3).

### 2.2. Analysis of Prevalence of M. bovis

**Figure 2:**
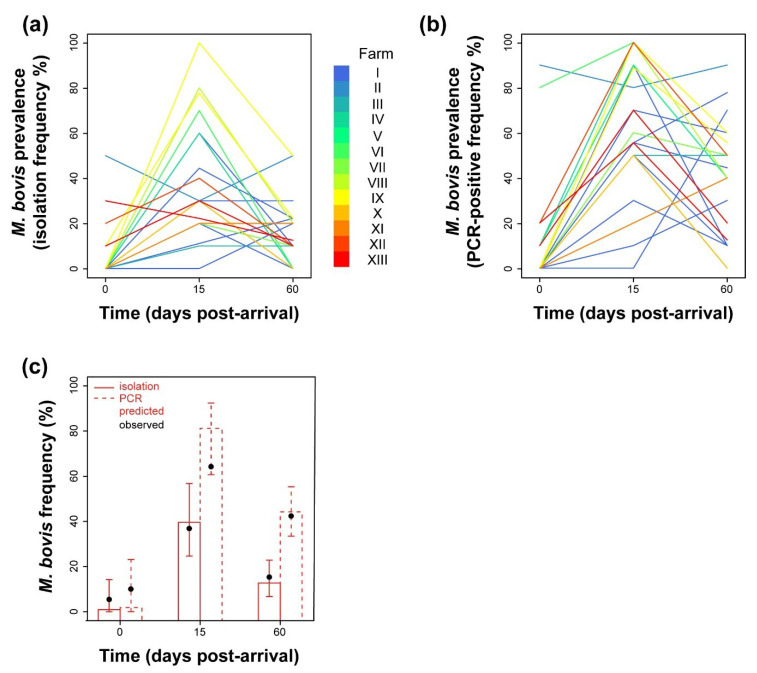
(**a**) Batch-related frequency of isolation of *M. bovis* analysed over time after arrival. Each line colour is depicted according to the identity of the stabling farm. (**b**) Batch-related frequency of *M. bovis*-specific PCR positives among bull batches analysed over time after arrival. (**c**) Model-predicted *M. bovis* prevalence, inferred at the population level and assessed from isolation (continuous line) and PCR (dashed line) data. Observed mean prevalence values from isolation and PCR data are depicted as solid black circles. Vertical lines correspond to the 95% CI of the predicted mean.

The frequency of *M. bovis* isolation clearly varied in a time-dependent fashion (Figure 2a). At arrival, 18 of 24 batches (75%) were negative for *M. bovis*, and 21 (87.5%) showed a prevalence lower than 10%. Such results were confirmed by PCR (Figure 2b): 17 of 24 batches (71%) were negative at arrival, and 20 (83%) showed a prevalence lower than 10%. 

With both approaches, we could clearly observe an increase in frequency at 15 p.a., followed by a general decrease 45 days later, which however largely varied in rate among farms/batches. The logistic models constructed confirmed such observations (Tables S4 and S6). At the population level, the probability of isolating *M. bovis* or testing bulls positive by PCR significantly depended on the time of sampling (Tables S5 and S7). The mean predicted *M. bovis* prevalence among newly introduced animals was estimated in the range of 1–2%, with upper confidence limits of 14% (isolation) and 23% (PCR) (Figure 2c). Such prevalence dramatically increased 15 days after introduction into stables, with an odds ratio of 73.4 for isolation (95% CI, 6.7–750, adjusted *p* = 0.015) and 213 for PCR (95% CI, 35–1901, adjusted *p* = 0.0001), to reach an estimated prevalence of approximately 40% (95% CI, 25–57%) in case of isolation and 81% (95% CI, 61–92%) according to PCR. At 60 days p.a., the estimated prevalence dropped to a lower level that differed with respect to the preceding one only when considering PCR-based frequency (adjusted *p* = 0.02). Environmental conditions did not show any predictive role in *M. bovis* prevalence (Tables S5 and S7). 

### 2.3. Analysis of Prevalence of M. dispar

**Figure 3:**
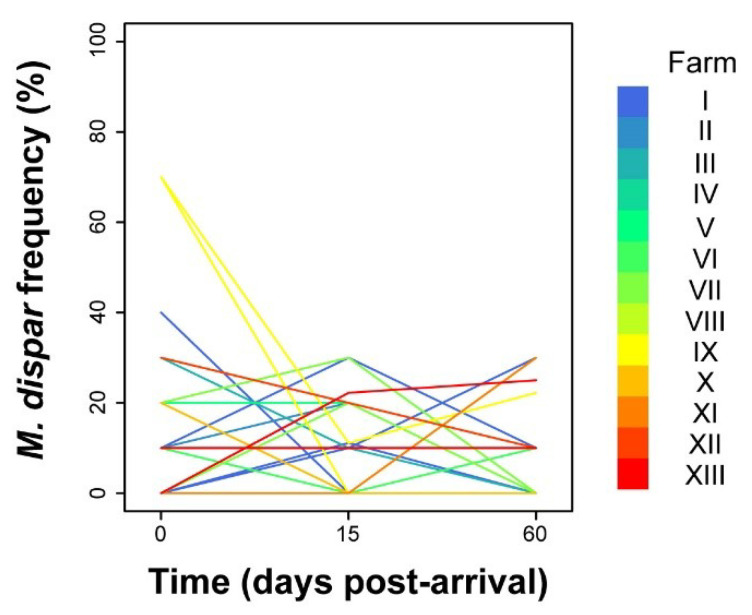
Batch-related frequency of isolation of *M. dispar* analysed over time after arrival. Each line colour is depicted according to the identity of the stabling farm.

Unlike *M. bovis*, the analysis of prevalence of *M. dispar* did not show any dependence on time, as shown by the batch trend lines (Figure 3) and the model we constructed (Tables S8 and S9). In fact, the mean predicted prevalence was estimated as constant with a value of 9.4% (95% CI, 6.7—13%). Similar to time, inclusion of seasonality did not increase the predictive power of the model (Table S9).

### 2.4. Analysis of Prevalence of M. bovirhinis

**Figure 4:**
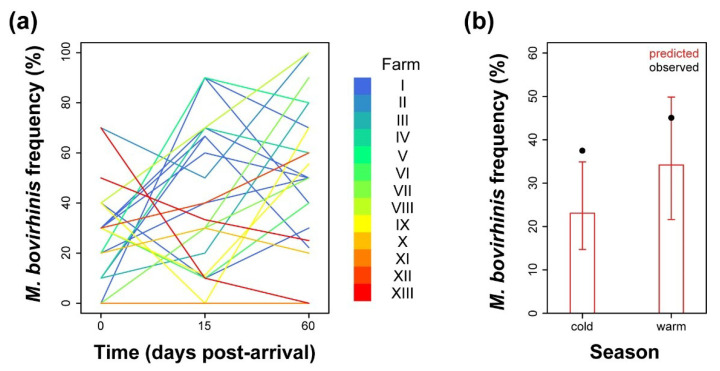
(**a**) Batch-related frequency of isolation of *M. bovirhinis* analysed over time after arrival. Each line colour is depicted according to the identity of the stabling farm. (**b**) Model-predicted *M. bovirhinis* prevalence inferred at the population level over arrival season (red). Observed mean prevalence values are depicted as solid black circles. Vertical lines correspond to the 95% CI of the predicted mean.

As already observed especially in the case of *M. bovis*, trend analysis of *M. bovirhinis* isolation over time post-arrival showed high variability among the sampled batches/farms (Figure 4a). Although there appeared to be an increase in prevalence over time, this was not significant (Table S11). Instead, we found that *M. bovirhinis* isolation probability depended on the stabling environmental conditions described by the variable “season” (Tables S10 and S11). In fact, the estimated mean prevalence of *M. bovirhinis* passed from 21.6% (95% CI, 12.9–33.9%), observed in the cold months of the year, to 33.1% (95% CI, 20–49.4%) in the warm season (Figure 4b), with an odds ratio of 1.8 (95% CI, 1.08–2.77). 

## 3. Discussion

The impact of BRD on cattle production is estimated to cause a decrease in mean carcass weight of at least 9 kg, leading to heavy losses of farmers’ incomes [7]. A better understanding of the spread of bovine mycoplasmas, involved in the BRD complex, may thus benefit practitioners by providing them with more comprehensive advice on how to control this significant economic and welfare problem [8]. The Italian bull production system is based on a singular approach typical of northeastern Italy and is believed, by local practitioners, to be exceptionally susceptible to BRD with a significant role played by mycoplasmas. However, the problems of this type of farming have not been well studied, leading to a poor understanding of the causes and risk factors of BRD.

The results of the present study showed that most nasal samples taken from bulls throughout the testing period were positive for organisms belonging to the *Mollicutes* class. Amplification of a fragment of the 16s rRNA gene followed by DGGE and profile comparison with reference strains led to their identification at species level (Table S1). In 71% of the cases, swabs were positive to *M. bovis* (19.1%), *M. dispar* (12.1%), and/or *M. bovirhinis* (39.8%) species, as pure or mixed cultures. In a previous work on Danish cattle farms [9], similar proportions of *M. bovirhinis* and *M. dispar* were detected, but *M bovis* was surprisingly absent. Our results showed a significant presence of *M. bovis* in the Italian bull meat sector with nearly a fifth of samples being positive, confirming other reports on the high prevalence of this mycoplasma in Britain [6], Ireland [10], France [8], and Canada [11].

In the present study, it appears evident that the majority of bulls arrived at the farm free of *M. bovis*, but within 2 weeks, its prevalence dramatically increased up to approximately 40% and 81% when tested by culture and *M. bovis*-specific PCR, respectively. Although high variability was observed at farm/batch level, our results showed that there was a rapid spread of *M. bovis* to the newly arrived bulls most likely from infected cattle already on the farm and/or possibly from the small number of infected imported bulls. In this regard, the phylogenetical typing of isolated strains could be useful to better understand the mechanism whereby *M. bovis* spreads among imported bulls, and future studies on that are strongly advised. The decrease in the percentage of infected cattle at 60 days p.a. indicates that some bulls overcame the infection to a point where it was no longer detectable in individual animals probably as a result of the host immune response mounted against this pathogen. Such a trend was seen in whatever diagnostic method used to detect *M. bovis-*positive bulls. 

In contrast to *M. bovis*, *M. dispar* prevalence did not follow a time-dependent behaviour. Much variability was observed among batches and farms (Figure 3), such that at the population level, it did not allow to reveal a common, statistically significant trend, suggesting a constant prevalence of 9.4%. Similarly, stabling animals in different seasons did not change the rate of spread of *M. dispar*. General unfavourable environmental conditions and/or the specific immune status of the bulls may account for the observed differences in the rate of spread between *M. bovis* and *M. dispar*. In our opinion, the low prevalence of *M. bovis* among incoming animals suggests the majority of these individuals may have been naïve to *M. bovis* infection, a condition that facilitated the spread of farm-resident *M. bovis* strains, exacerbated by the close contact with infected older bulls in overcrowded conditions. In contrast, the higher *M. dispar* prevalence observed already on arrival may indicate that the immune systems of the incoming animals were already primed to this mycoplasma species, providing a protective shield against *M. dispar* infection and spread. Alternatively, an unfavourable environment and breeding conditions may have limited the spread. It is also possible that the high prevalence of *M. bovis* may have competitively excluded the colonisation of *M dispar* although evidence is needed to support this. 

Similarly to *M. dispar*, *M. bovirhinis* prevalence showed high variability among batches and farms, such that we could not statistically define a common trend over time. However, we observed a significant effect of environmental conditions brought about by seasonality, with higher prevalence associated with warmer conditions. This trait seems to be specific for this species as it was not observed with *M. bovis* and *M. dispar*. *M. bovirhinis* is not considered a primary pathogen and, although it is one of the most commonly occurring species in respiratory diseases [6], it can also be frequently isolated from healthy or asymptomatic animals, where it may be considered part of the natural bacterial flora. The decrease in prevalence of *M. bovirhinis* seen in the winter months may be due to the preferential colonisation of respiratory pathogens, including *M. bovis* and *M. dispar* [12,13,14], when cattle are more susceptible. Alternatively, such an association may derive from spurious effects given by hidden confounding variables. 

In conclusion, our results showed that the Italian fattening bull system creates an ideal environment for the spread and diffusion of *Mollicutes* and, more specifically, of *M. bovis*. The spread of the latter did not seem to be related to the health status of the new bulls; in fact, the high circulation of *M. bovis* is localised during the first weeks after arrival. Most likely, the spread was related to the presence of older infected bulls that provided the source of infection, possibly a dominant farm-specific *M. bovis* strain, to the newly imported bulls as previously reported [8]. A similar situation is seen in other livestock sectors, such as multiage layers hens flocks where the spread of mycoplasma from older birds can cause economic losses in the newest flocks [15,16]. This kind of problem has been controlled in the poultry industry by “all in, all out” systems stocked with *Mycoplasma*-free or by vaccination and could be applied with specific modifications to the bull meat sector studied here. The newly acquired knowledge of *M. bovis* diffusion dynamics from this study will enable better management of BRD, focusing on the herd management, such as improving ventilation and other husbandry techniques.

## 4. Materials and Methods 

### 4.1. Animals

In this study, we longitudinally analysed 24 different male cattle batches, imported in 2011–2013 and stabled into 13 different fattening farms (identified as I–XIII) of Northern Italy. Most batches consisted of 54 heads of cattle, in large part imported from France. The capacity of the selected farms differed among each other, ranging from 400 to 1500 bulls per farm. For each batch, 10 bulls were randomly selected and sampled for the entire period of the study, with the exception of 7 animals that were lost during the observational period due to mortality or slaughtering (Table S12). Two deep nasal swabs, one for *M. bovis* PCR and the other for *Mollicutes* isolation, were taken from each animal on arrival, and at 15 and 60 days after arrival. A total of 711 samples were collected: 240 on arrival, 238 at the second, and 233 at the third sampling. 

### 4.2. Mollicute Cultivation 

To ensure *Mollicutes* vitality, immediately after sampling, swabs were immersed into 2 mL of *Mycoplasma* liquid medium (ML; Mycoplasma Experience Ltd., Bletchingley, UK) and maintained at 4 °C until arrival at the laboratory. Mycoplasma cultivation and isolation were then performed in ML and PPLO (Pleuro-Pneumonia like Organisms) broth media. Briefly, the inoculated cultures were incubated at 37 °C with 5% CO_2_ for at least 7 days. The broths were checked daily up to 14 days to detect any change in colour or turbidity. Broths that showed any change were immediately inoculated onto a plate of semisolid *Mycoplasma* agar medium (MS; Mycoplasma Experience). Alternatively, broths that did not show any change were plated onto agar medium at the end of the observation period. If no colonies grew after 14 days, the sample was considered negative. 

### 4.3. Mycoplasma Identification

To identify the species of the different *Mollicutes* grown in broth media, DNA was extracted with the Maxwell 16 LEV Blood DNA kit and Maxwell 16 Instrument following the manufacturer’s instructions (Promega), amplified by a 16S-rDNA-targeting PCR and analysed by denaturing gradient gel electrophoresis (DGGE), following a previously reported protocol [17]. Identification of the different *Mollicutes* genera and species was carried out by direct comparison of the lane of interest with the profile of reference strains. To investigate the presence of *M. bovis* DNA on the collected swabs, total DNA was extracted from an aliquot of the relative transport medium, amplified by a *M. bovis*-specific PCR protocol [18] and analysed by electrophoresis in 1% agar gel. 

### 4.4. Statistical Analysis

The statistical analysis of this study was conducted under R environment [19]. The prevalence of organisms belonging to the *Mollicutes* class and to the species *M. bovis*, *M. dispar*, and *M. bovirhinis* was analysed according to a longitudinal framework, in which the same animals were repeatedly sampled along time post arrival. In addition, the potential correlation among observations from the same animals (coded by the variable “ID”) and from bulls belonging to the same batch (“batch” variable) or farm (“farm” variable) was considered. For such reasons, we decided to construct logistic mixed effects (hierarchical) models to predict bulls’ positivity to each of the 4 considered organisms. For all models, we first determined the correlation structure that best suited to the observed data. Random intercept models were constructed, assuming as random effects the covariates “ID”, “farm”, and “batch” alone or in nestling combinations. Random intercept and slope models were then evaluated, adding a random slope described by the categorical covariate “time” (time post arrival) to the previously selected random intercept model. In all cases, the best-fitting correlation structure was described by a random slope associated to the covariate “time” and a random intercept expressed by the grouping variable “farm”. At the population level, in addition to the covariate “time”, we tested the descriptive variable “season”, coded as “cold” if the bulls were stabled between November and March and “warm” otherwise. The significance of both random and fixed effects variables was estimated by repeated likelihood ratio tests. All models but the ones predicting the probability of isolating *M. bovis* and *M. dispar* were estimated with the function *glmer* of the *lme4* package [20], applying a maximum likelihood with Laplace approximation and “bobyqa” optimisation for convergence. In the case of the models describing *M. bovis* and *M. dispar* prevalence from isolation, the aforementioned approach led to singular fits, in which some components of the variance–covariance matrix were estimated as zero. To overcome this problem, we employed the function *bglmer* of the package *blme* [21] that allows obtaining inferences based on a penalised maximum likelihood with priors for the covariance matrix of the random effects following a Wishart distribution. Multiple comparisons were performed with the function *pairs* of the package *emmeans*, applying Tukey’s *p* value adjustment method [22].

## Supplementary Materials

The following are available online at https://www.mdpi.com/2076-0817/9/7/537/s1, Table S1: Species and genera of the *Mollicutes* class isolated from the analysed nasal swabs. Table S2: Parameter estimates of the logistic mixed effects model analysing the isolation frequency of organisms of the *Mollicutes* class. Table S3: Analysis of deviance table (type II likelihood ratio tests) of the full model relating the isolation frequency of organisms of the *Mollicutes* class to the variables time and season.. Table S4: Parameter estimates of the logistic mixed effects model analyzing the frequency of *M. bovis* isolation. Table S5: Analysis of deviance table (type II likelihood ratio tests) of the full model relating the frequency of isolation of *M. bovis* to the variables time and season. Table S6: Parameter estimates of the logistic mixed effects model analyzing the frequency of *M. bovis*-specific PCR positives. Table S7: Analysis of deviance table (type II likelihood ratio tests) of the full model relating the frequency of *M. bovis*-specific PCR positives to the variables time and season. Table S8: Parameter estimates of the logistic mixed effects model analyzing the frequency of *M. dispar* isolation. Table S9: Analysis of deviance table (type II likelihood ratio tests) of the full model relating the frequency of isolation of *M. dispar* to the variables time and season. Table S10: Parameter estimates of the logistic mixed effects model analyzing the frequency of *M. bovirhinis* isolation. Table S11: Analysis of deviance table (type II likelihood ratio tests) of the full model relating the frequency of isolation of *M. bovirhinis* to the variables time and season. Table S12: Data structure description.
