# Supplementary material for: Infection Dynamics of Mycoplasma bovis and Other Respiratory Mycoplasmas in Newly Imported Bulls on Italian Fattening Farms
Source: Pathogens. 2020 Jul 4;9(7):537. doi: 10.3390/pathogens9070537 (PMC7399803; doi:10.3390/pathogens9070537)
Supplement: Supplementary file 1 [file pathogens-09-00537-s001.pdf]

Table S1. Species and genera of the *Mollicutes* class isolated from the analyzed nasal swabs.

**Isolates**	# **positives (%)**	# **pure cultures (%)**
M. bovirhinis	283 (39.8)	130 (18.3)
M. bovis	136 (19.1)	47 (6.6)
M. dispar	86 (12.1)	51 (7.2)
M. arginini	40 (5.6)	7 (1)
M. alkalescens	26 (3.7)	9 (1.3)
M. ovipneumoniae	5 (0.7)	4 (0.6)
M. fermentans	3 (0.4)	2 (0.3)
Ureaplasma spp.	66 (9.3)	4 (0.6)
A. laidlawii	16 (2.2)	10 (1.4)

Table S2: Parameter estimates of the logistic mixed effects model analyzing the isolation frequency of organisms of the *Mollicutes* class.

Fixed Effects

Predictors	*Log-Odds*	*95% CI*	*p* *
(Intercept)	-0.09	-0.65 – 0.61	0.830
time [day 15]	1.53	0.80 – 2.35	0.001
time [day 60]	1.80	0.66 – 2.73	0.001

Random Effects

Groups	*Predictors*	*Std.Dev*	*Corr.*	
farm	(Intercept)	1.27		
	time [day 15]	1.30	-0.46	
	time [day 60]	1.56	-0.49	0.84

* Wald's test *p* value

Table S3: Analysis of deviance table (type II likelihood ratio tests) of the full model relating the isolation frequency of organisms of the *Mollicutes* class to the variables time and season.

**Fixed Effects**				
Predictors	*npar*	*AIC*	X^2	*p*
time	2	759.68	9.50	0.009
season	1	752.52	0.34	0.56
**Random Effects**				
Predictors	*npar*	*AIC*	X^2	*p*
time∣farm	6	820.55	78.37	7.75×10^{-15}

Legend: *npar* = number of parameters associated to the relative predictor; *AIC* = Akaike's information criterion value observed upon dropping of the relative predictor; X^2 = likelihood ratio test statistic; p = likelihood ratio test *p* value.

Table S4: Parameter estimates of the logistic mixed effects model analyzing the frequency of *M. bovis* isolation

Fixed Effects

Predictors	*Log-Odds*	*95% CI*	*p* *
(Intercept)	-4.72	-6.68 – -2.58	0.002
time [day 15]	4.30	1.90 – 6.62	0.005
time [day 60]	2.79	0.79 – 4.65	0.049

Random Effects

Groups	*Predictors*	*Std.Dev*	*Corr.*	
farm	(Intercept)	2.94		
	time [day 15]	3.20	-0.94	
	time [day 60]	2.40	-0.97	0.95

* Wald's test *p* value

Table S5: Analysis of deviance table (type II likelihood ratio tests) of the full model relating the frequency of isolation of *M. bovis* to the variables time and season.

**Fixed Effects**				
Predictors	*npar*	*AIC*	X^2	*p*
time	2	600.69	13.24	0.001
season	1	589.69	0.25	0.62
**Random Effects**				
Predictors	*npar*	*AIC*	X^2	*p*
time∣farm	6	619.79	40.35	3.89×10^{-7}

Legend: *npar* = number of parameters associated to the relative predictor; *AIC* = Akaike's information criterion value observed upon dropping of the relative predictor; X^2 = likelihood ratio test statistic; p = likelihood ratio test *p* value.

Table S6: Parameter estimates of the logistic mixed effects model analyzing the frequency of *M. bovis*-specific PCR positives.

**Fixed Effects**				
Predictors		*Log-Odds*	*95% CI*	*p* *
(Intercept)		-3.89	-6.19 – -2.05	0.005
time [day 15]		5.36	3.55 – 7.55	<0.001
time [day 60]		3.65	1.90 – 6.27	0.006
**Random Effects**				
Groups	*Predictors*	*Std.Dev*	*Corr.*	
farm	(Intercept)	3.53		
	time [day 15]	2.79	-0.91	
	time [day 60]	3.31	-0.99	0.93

* Wald's test *p* value

Table S7: Analysis of deviance table (type II likelihood ratio tests) of the full model relating the frequency of *M. bovis*-specific PCR positives to the variables time and season.

**Fixed Effects**				
Predictors	*npar*	*AIC*	X^2	*p*
time	2	708.25	20.5	3.60×10^{-5}
season	1	689.85	0.061	0.81
**Random Effects**				
Predictors	*npar*	*AIC*	X^2	*p*
time∣farm	6	780.14	100.35	2.12×10^{-19}

Legend: *npar* = number of parameters associated to the relative predictor; *AIC* = Akaike's information criterion value observed upon dropping of the relative predictor; X^2 = likelihood ratio test statistic; p = likelihood ratio test *p* value.

Table S8: Parameter estimates of the logistic mixed effects model analyzing the frequency of *M. dispar* isolation

Fixed Effects

Predictors	*Log-Odds*	*95% CI*	*p* *
(Intercept)	-2.27	-2.63 – -1.91	> 0.001

Random Effects

Groups	*Predictors*	*Std.Dev*	*Corr.*	
farm	(Intercept)	1.28		
	time [day 15]	1.55	-0.89	
	time [day 60]	1.79	-0.93	0.75

* Wald's test *p* value

Table S9: Analysis of deviance table (type II likelihood ratio tests) of the full model relating the frequency of isolation of *M. dispar* to the variables time and season.

**Fixed Effects**				
Predictors	*npar*	*AIC*	X^2	*p*
time	2	514.84	1.93	0.38
season	1	517.36	2.44	0.12
**Random Effects**				
Predictors	*npar*	*AIC*	X^2	*p*
time|farm	6	517.77	12.86	0.04

Legend: *npar* = number of parameters associated to the relative predictor; *AIC* = Akaike's information criterion value observed upon dropping of the relative predictor; X^2 = likelihood ratio test statistic; p = likelihood ratio test *p* value.

Table S10: Parameter estimates of the logistic mixed effects model analyzing the frequency of *M. bovirhinis* isolation

Fixed Effects

Predictors	*Log-Odds*	*95% CI*	*p* *
(Intercept)	-1.29	-1.69 – 0.73	> 0.001
season [warm]	0.59	0.08 – 1.02	0.014

Random Effects

Groups	*Predictors*	*Std.Dev*	*Corr.*	
farm	(Intercept)	0.90		
	time [day 15]	1.55	-0.51	
	time [day 60]	2.50	-0.04	0.68

* Wald's test *p* value

Table S11: Analysis of deviance table (type II likelihood ratio tests) of the full model relating the frequency of isolation of *M. bovirhinis* to the variables time and season.

**Fixed Effects**				
Predictors	*npar*	*AIC*	X^2	*p*
time	2	858.51	5.84	**0.**054
season	1	860.64	5.96	**0.**014
**Random Effects**				
Predictors	*npar*	*AIC*	X^2	*p*
time∣farm	6	921.60	76.93	1.54×10^{-14}

Legend: *npar* = number of parameters associated to the relative predictor; *AIC* = Akaike's information criterion value observed upon dropping of the relative predictor; X^2 = likelihood ratio test statistic; p = likelihood ratio test *p* value.

Table S12: Data structure description

Farm	batches	season*	Bulls analyzed at		
			0 days p.a.	15 days p.a.	60 days p.a.
I	1	warm	10	10	10
	2	warm	10	10	10
	3	warm	10	10	10
	4	warm	10	10	10
	5	warm	10	9	9
	6	warm	10	10	10
	7	cold	10	10	10
	8	cold	10	10	9
	9	cold	10	10	9
II	1	cold	10	10	10
III	1	cold	10	10	10
IV	1	cold	10	10	10
V	1	cold	10	10	10
VI	1	cold	10	10	10
VII	1	warm	10	10	10
	2	cold	10	10	10
VIII	1	warm	10	10	10
IX	1	cold	10	10	9
	2	cold	10	10	9
X	1	cold	10	10	10
XI	1	cold	10	10	10
XII	1	cold	10	10	10
XIII	1	warm	10	9	8
	2	warm	10	10	10

p.a.: *post* arrival

* Season describes the environmental conditions at the arrival: “cold” if the bulls were stabled between November and March, “warm” otherwise
